# Special Issue: “Pediatric Orthopedic Malignancy: Types, Symptoms, and Treatment”

**DOI:** 10.3390/children10091545

**Published:** 2023-09-13

**Authors:** Hisaki Aiba, Shinji Miwa, Hideki Murakami, Hiroaki Kimura

**Affiliations:** 1 Department of Orthopedic Surgery, Graduate School of Medical Sciences, Nagoya City University, Nagoya 467-8601, Japan; hmuraka@med.nagoya-cu.ac.jp (H.M.); hiroaki030301@yahoo.co.jp (H.K.); 2 Department of Orthopedic Surgery, Graduate School of Medical Science, Kanazawa University, Kanazawa 920-8640, Japan; smiwa001@yahoo.co.jp

## 1. Main Pediatric Orthopedic Malignancies: Types and Symptoms

Pediatric orthopedic malignancies are extremely rare and require appropriate diagnosis and treatment by a multidisciplinary team [1]. There are two major primary malignant bone tumors in children: osteosarcoma and Ewing sarcoma [2]. Osteosarcoma is composed of spindle cell neoplasms that produce osteoid [1], and usually occurs in the metaphyses of the long bones in the knee, the proximal humerus, and the proximal femur [3]. Usually, a short history (weeks to months) of a painful or palpable mass precedes the diagnosis of osteosarcoma [4]. Pathological fractures occur in 10–15% of patients [5]. Although localized disease has a five-year survival rate of >80% with combination chemotherapy and complete resection [6], patients with pulmonary metastases at initial presentation have a poor prognosis [6]. Surgery for the primary site is an essential part of the treatment of patients with osteosarcoma [7]. Typically, chemotherapy is administered before and after surgery, using a combination therapy that includes methotrexate, cisplatin, and doxorubicin [7]. On the other hand, the second-line therapy options for relapsed or metastatic osteosarcoma remain limited [8,9].

Ewing sarcoma is the second most common primary bone tumor in children and adolescents [6]. The clinical presentation of Ewing sarcoma is often nonspecific, with the most common symptoms being pain, swelling, and general discomfort [10]. Histologically, the tumor is composed of small round cells, and mainly caused by the chromosomal translocation of *EWSR1* on chromosome 22; t(11;22)(q24;q12) or t(21;22)(q22;q12) [11,12,13]. The five-year overall survival rates are approximately 70–80% and 20–30% for patients with localized Ewing sarcoma and metastatic disease, respectively [2]. The primary therapy for Ewing sarcoma typically involves a combination treatment with vincristine, doxorubicin, and cyclophosphamide, alternated with ifosfamide and etoposide, both before and after local therapy [14]. The choice of local therapy for Ewing sarcoma depends on the anatomical site and the feasibility of surgery. When wide resection with negative margin is not possible or will cause serious morbidity, radiotherapy or both surgery and radiotherapy, are considered as an acceptable alternative to local control [15]. For patients with relapsed or metastatic Ewing sarcoma, second-line therapies such as cyclophosphamide + topotecan [16], irinotecan + temozolomide ± vincristine [17,18], or docetaxel + gemcitabine [19] are commonly employed.

Soft tissue sarcomas account for approximately 8% of all malignancies in childhood and adolescence [20]. Approximately half of the cases are rhabdomyosarcoma, while the remainder are various entities, including infantile fibrosarcoma, rhabdoid tumor, synovial sarcoma, liposarcoma, leiomyosarcoma, and undifferentiated pleomorphic sarcoma [20]. Rhabdomyosarcoma is a family of sarcomas with morphological and/or immunophenotypic evidence of skeletal muscle differentiation and comprises embryonal, alveolar, spindle cell/sclerosing, and pleomorphic types. They present with site-specific symptoms, particularly the alveolar type, which is highly aggressive and typically presents as a rapidly growing soft tissue mass, occasionally with symptoms mimicking leukemia (e.g., fatigue, fever, and unexplained weight loss) [21,22]. Embryonal types generally have a favorable prognosis, whereas alveolar types are more aggressive [23]. The treatment for patients is determined by risk-based therapy, typically with a combination of chemotherapy and site-specific surgery and/or radiotherapy [24]. The European Paediatric Soft Tissue Sarcoma Study Group [25,26] and the North American Soft Tissue Sarcoma Committee of the Children’s Oncology Group (COG) [27,28] have both made efforts to establish risk-adapted standards of individual care for patients with rhabdomyosarcoma. 

## 2. Advancements in the Treatment of Pediatric Orthopedic Malignancies

Pediatric orthopedic malignancies should be treated by a multidisciplinary team for optimal surgical outcomes and patient care [29]. For most cases, surgical resection of the primary tumor combined with chemotherapy and/or radiotherapy is required [30,31]. Wide resection with negative margins is the cornerstone of the surgical treatment of the primary tumor [32]. To improve the surgical treatment, novel technologies, such as three-dimensional (3D) printing and augmented reality, offer promising solutions [33]. Patient-specific cutting guides and instruments created using 3D printing technology are valuable, particularly for pelvic bone tumors, aiding in precise resection and reconstruction [34]. These techniques help achieve accurate resection with safe margins and improve oncologic outcomes [34]. Moreover, surface modification techniques for implants coated with silver, iodine, or gentamicin have been used in the orthopedic field and are expected to reduce the infection risk [35,36]. Further research is required to understand their impact on infection prevention and implant durability.

In contrast, systemic therapy remains a challenge for patients with unresectable or metastatic pediatric orthopedic malignancies. The rarity and heterogeneity of the malignancies have hindered advancements in drug development and management [20]. However, recent breakthroughs in diagnostic molecules and targeted therapies for pediatric orthopedic malignancies have led to more accurate diagnoses and the emergence of novel therapeutic targets [30].

In terms of the genomic abnormalities in osteosarcoma, copy number alterations and chromosomal instability are commonly reported [37]. The loss of *TP53* and *RB1*, as well as the amplification of *CDK4*, are widely recognized as key factors in the pathogenesis of osteosarcoma [37]. Additionally, multi-kinase inhibitors have shown applicability in suppressing hyper-activated receptors such as FGFR, VEGFR, and c-KIT [38,39]. Furthermore, some patients with osteosarcoma exhibit a high level of HER-2 protein expression [24]. Trastuzumab deruxtecan was investigated in a phase 2 study for patients with HER2-positive, relapsed, unresectable osteosarcoma; however, only one of nine patients achieved a response by reaching 24 weeks of event-free survival, and the estimated response rate was 11.1% (NCT04616560) [40].

Similarly, the genetic background of Ewing sarcoma can serve as a biomarker for treatment selection and as a target for molecular therapies [37]. Ewing sarcoma has been reported to exhibit copy number alterations or mutations in genes such as *STAG2*, *TP53*, and *CDKN2A* [37,41]. Utilizing various biomarkers, including *STAG2* mutation, *TP53* mutation status, and baseline circulating tumor DNA burden, the Bone Tumor Committee of the COG aims to stratify standard treatment [37]. This approach aims to reduce the intensity of standard chemotherapy for low-risk patients while incorporating targeted therapies for high-risk patients [37]. Moreover, *EWS-FLI1*, an oncogenic transcription factor, downregulates intrinsic CDK inhibitors, playing a crucial role in cell cycle regulation [42]. A randomized, open-label, phase 2 study, as part of the CAMPFIRE master protocol, will assess the addition of abemaciclib, a selective *CDK4/6* inhibitor, to the standard treatment with irinotecan + temozolomide for the treatment of relapsed/refractory Ewing sarcoma [42]. 

Surgical treatment for patients with metastases remains controversial. A systematic review of metastatic soft tissue sarcoma suggested that fewer metastases and an achievement of complete metastasectomy were related to better survival after metastasectomy [43]. Moreover, less-invasive local therapies, such as radiofrequency ablation, are useful alternatives to metastasectomy [44]. AOST2031 will compare open surgery and minimally invasive surgery for pulmonary metastatic osteosarcoma to evaluate thoracic event-free survival and determine the superior surgical approach, thereby affecting curative therapy decisions for patients with metastases [45].

Research about the mechanisms of progression of pediatric orthopedic malignancies including proliferation, angiogenesis, migration, invasion, microenvironment, and antitumor immune system activation has progressed [30]. Novel anticancer agents, including immune checkpoint inhibitors and molecular targeting agents, have been developed [46]. Various immunotherapies have been developed to treat malignant tumors [47]. Historically, bone and soft tissue sarcomas were the first tumors to be treated with immunotherapy. Coley reported that the injection of inactivated bacteria induced tumor shrinkage and increased survival rates [48]. Recently, the efficacies of various immunotherapies, including dendritic-cell-based immunotherapy, chimeric antigen receptor-modified T cell therapy, adoptive T cell therapy, and immune check inhibitors, have been investigated in several clinical trials [49,50,51,52,53]. For instance, a combination therapy with cabozantinib, nivolumab, and ipilimumab for metastatic soft tissue sarcoma lacking translocation was reported in a randomized phase 2 clinical trial (NCT04551430) [54]. The trial involved 69 patients receiving the combination and 36 patients receiving cabozantinib alone with a crossover [54]. The combination demonstrated a higher overall disease control rate (80% vs. 42%, cabozantinib with nivolumab and ipilimumab, and cabozantinib alone, respectively), and improved progression-free survival (5.4 vs. 3.8 months, cabozantinib with nivolumab and ipilimumab and cabozantinib alone, respectively) [54]. Although these studies have proven treatment efficacy in a limited number of patients, further research on the therapeutic targets and mechanisms of pediatric orthopedic malignancy progression may improve immunotherapy outcomes.

## 3. Conclusions

Pediatric orthopedic malignancies are rare and require a correct diagnosis and multimodal treatment. In this Special Issue “Pediatric Orthopedic Malignancy: Types, Symptoms, and Treatment”, we will focus on the latest clinical/basic studies and the development of technologies to improve the surgical treatment of pediatric orthopedic malignancies.
